# Supplementary material for: A model-based cost-utility analysis of an automated notification system for deteriorating patients on general wards
Source: PLoS One. 2024 May 2;19(5):e0301643. doi: 10.1371/journal.pone.0301643 (PMC11065309; doi:10.1371/journal.pone.0301643)
Supplement: S1 Fig — (DOCX) [file pone.0301643.s002.docx]

## **S13 Fig. Cost-effectiveness plane for base-case and all subgroup analyses.**

Interpreting net benefit on the cost-effectiveness plane: Indifference curves of identical slope are plotted through each strategy, with the slope representing the £20,000 per quality-adjusted life-year (QALY), threshold of decision making the UK NHS. The points where each indifference curve intersects the horizontal and vertical axes (⯁) represents the net health benefit (NHB) and the net monetary benefit (NMB), respectively, for all strategies on the respective indifference curve. Strategies on lower indifference curves are relatively more cost-effective than strategies on higher indifference curves. The cost and benefit for each strategy is represented by the ICER (●).

*Solid grey line = £20,000/QALY threshold, all strategies below this line are considered cost-effective*

*
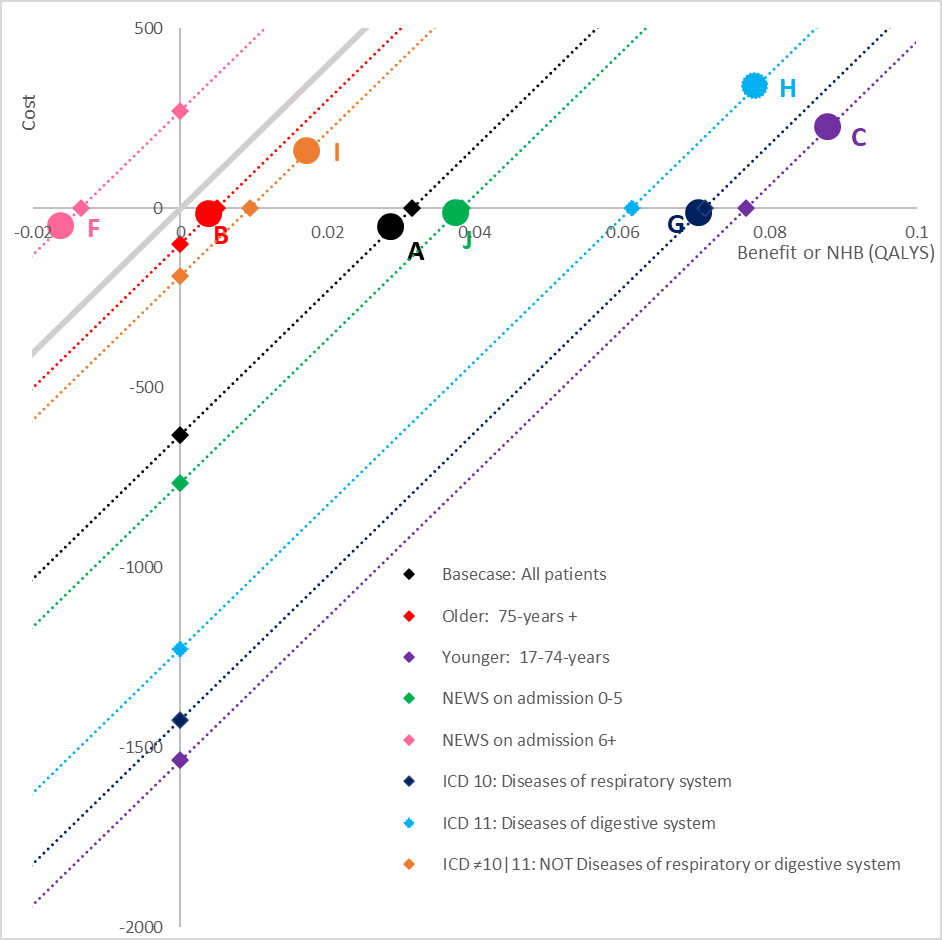
*
