# Supplementary figures and images for: A model-based cost-utility analysis of an automated notification system for deteriorating patients on general wards
Source: PLoS One. 2024 May 2;19(5):e0301643. doi: 10.1371/journal.pone.0301643 (PMC11065309; doi:10.1371/journal.pone.0301643)

## **S14 Fig. Cost-effectiveness plane for base-case and all subgroups by age.**

*
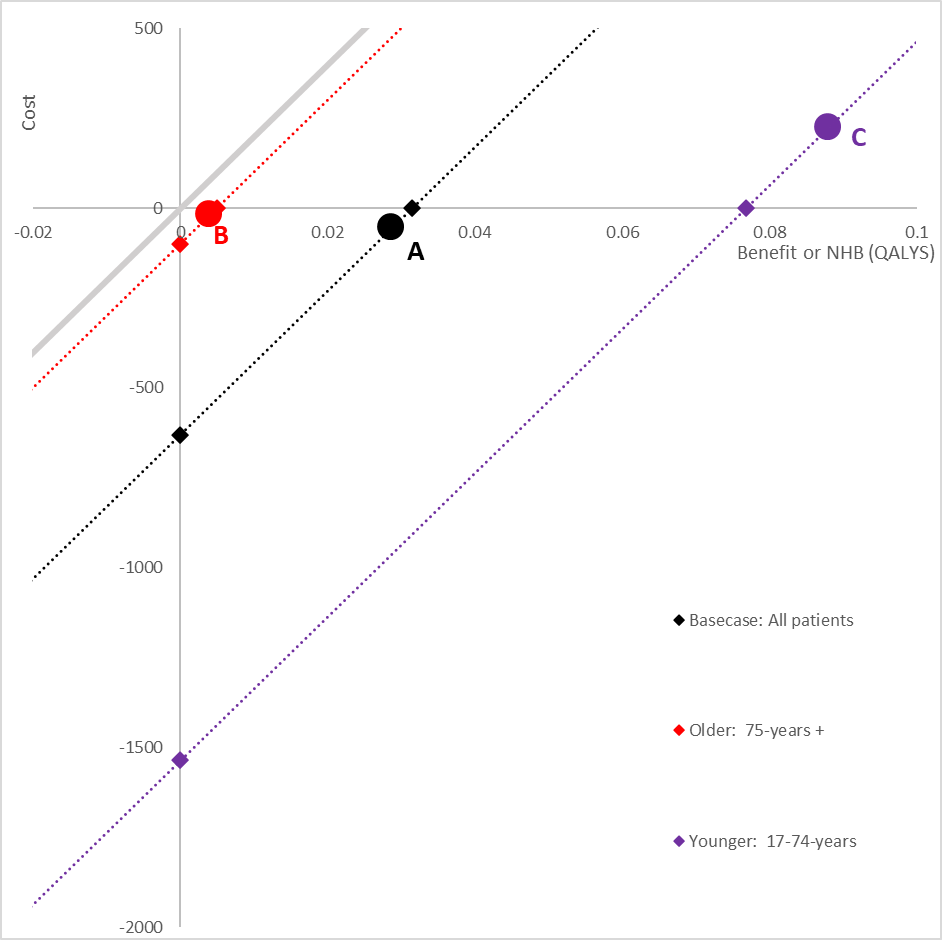
*

Supplement: S2 Fig — (DOCX) [file pone.0301643.s003.docx]

## **S15 Fig. Cost-effectiveness plane for base-case and all subgroups by NEWS on admission.**

*
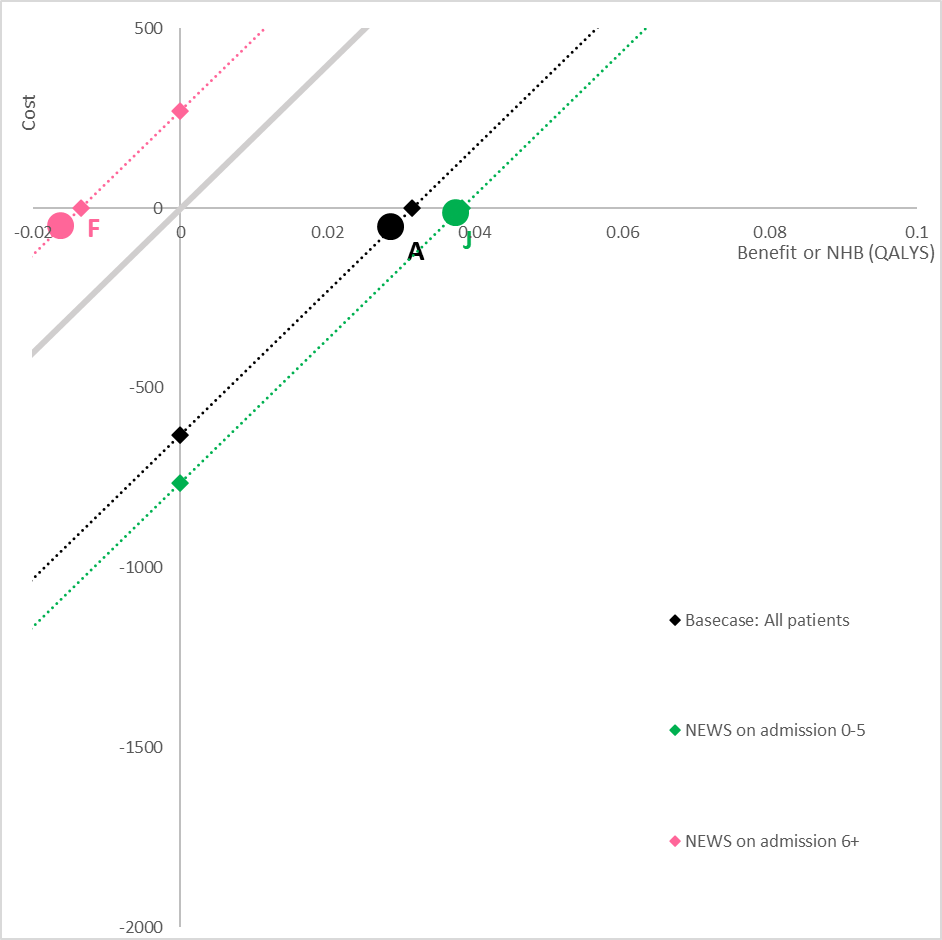
*

Supplement: S3 Fig — (DOCX) [file pone.0301643.s004.docx]

## **S16 Fig. Cost-effectiveness plane for base-case and all subgroups by Primary ICD code.**

*
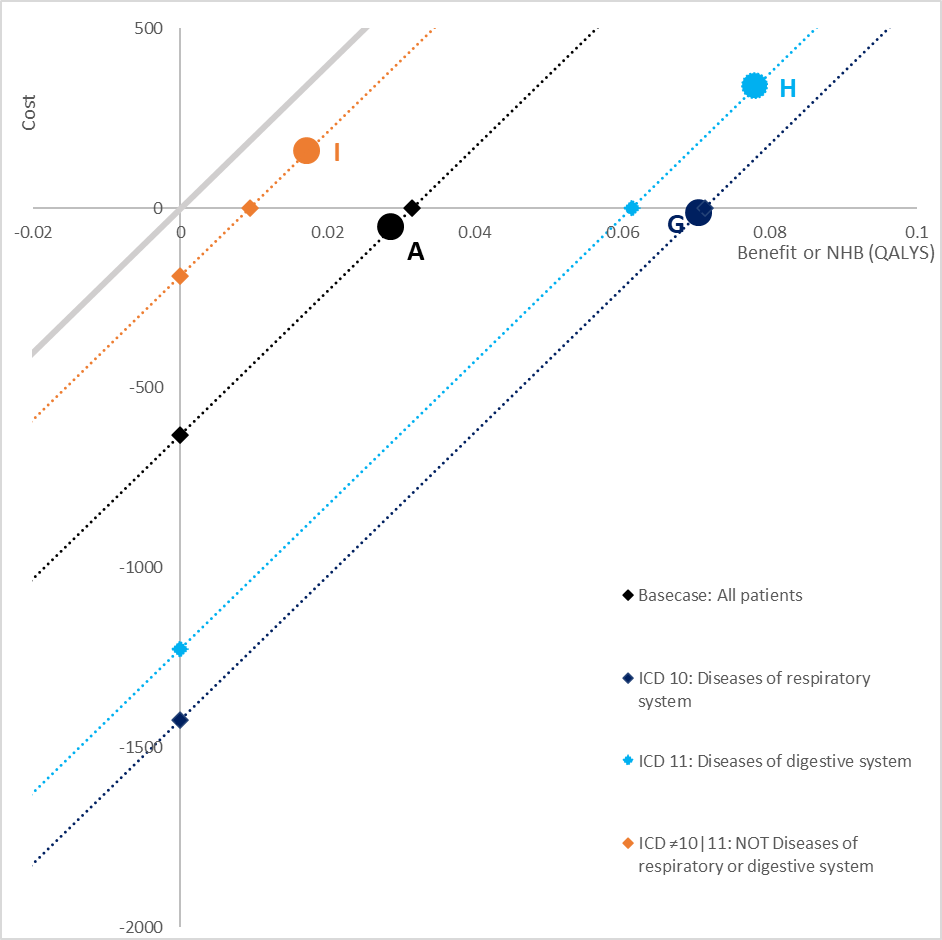
*

Supplement: S4 Fig — (DOCX) [file pone.0301643.s005.docx]
