# Supplementary material for: A model-based cost-utility analysis of an automated notification system for deteriorating patients on general wards
Source: PLoS One. 2024 May 2;19(5):e0301643. doi: 10.1371/journal.pone.0301643 (PMC11065309; doi:10.1371/journal.pone.0301643)
Supplement: S1 Table — (DOCX) [file pone.0301643.s006.docx]

**S2 Table. NHS Reference Costs Ward 1 (gastroenterology) inclusion and exclusion.**

*Following clinical review (CS 05/05/22) the following currencies were considered plausible and included in the frequency weighted average calculation.*

| **Currency** | **Currency Description** |
| --- | --- |
|  |  |
| FD01A | Gastrointestinal Infections with Multiple Interventions, with CC Score 4+ |
| FD01B | Gastrointestinal Infections with Multiple Interventions, with CC Score 0-3 |
| FD01C | Gastrointestinal Infections with Single Intervention, with CC Score 5+ |
| FD01D | Gastrointestinal Infections with Single Intervention, with CC Score 2-4 |
| FD01E | Gastrointestinal Infections with Single Intervention, with CC Score 0-1 |
| FD01F | Gastrointestinal Infections without Interventions, with CC Score 8+ |
| FD01G | Gastrointestinal Infections without Interventions, with CC Score 5-7 |
| FD01H | Gastrointestinal Infections without Interventions, with CC Score 2-4 |
| FD01J | Gastrointestinal Infections without Interventions, with CC Score 0-1 |
| FD02A | Inflammatory Bowel Disease with Multiple Interventions, with CC Score 3+ |
| FD02B | Inflammatory Bowel Disease with Multiple Interventions, with CC Score 0-2 |
| FD02C | Inflammatory Bowel Disease with Single Intervention, with CC Score 4+ |
| FD02D | Inflammatory Bowel Disease with Single Intervention, with CC Score 0-3 |
| FD02E | Inflammatory Bowel Disease without Interventions, with CC Score 5+ |
| FD02F | Inflammatory Bowel Disease without Interventions, with CC Score 3-4 |
| FD02G | Inflammatory Bowel Disease without Interventions, with CC Score 1-2 |
| FD02H | Inflammatory Bowel Disease without Interventions, with CC Score 0 |
| FD03A | Gastrointestinal Bleed with Multiple Interventions, with CC Score 5+ |
| FD03B | Gastrointestinal Bleed with Multiple Interventions, with CC Score 0-4 |
| FD03C | Gastrointestinal Bleed with Single Intervention, with CC Score 8+ |
| FD03D | Gastrointestinal Bleed with Single Intervention, with CC Score 5-7 |
| FD03E | Gastrointestinal Bleed with Single Intervention, with CC Score 0-4 |
| FD03F | Gastrointestinal Bleed without Interventions, with CC Score 9+ |
| FD03G | Gastrointestinal Bleed without Interventions, with CC Score 5-8 |
| FD03H | Gastrointestinal Bleed without Interventions, with CC Score 0-4 |
| FD04A | Nutritional Disorders with Interventions, with CC Score 2+ |
| FD04B | Nutritional Disorders with Interventions, with CC Score 0-1 |
| FD04C | Nutritional Disorders without Interventions, with CC Score 6+ |
| FD04D | Nutritional Disorders without Interventions, with CC Score 2-5 |
| FD04E | Nutritional Disorders without Interventions, with CC Score 0-1 |
| FD05A | Abdominal Pain with Interventions |
| FD05B | Abdominal Pain without Interventions |
| FD10A | Non-Malignant Gastrointestinal Tract Disorders with Multiple Interventions, with CC Score 8+ |
| FD10B | Non-Malignant Gastrointestinal Tract Disorders with Multiple Interventions, with CC Score 5-7 |
| FD10C | Non-Malignant Gastrointestinal Tract Disorders with Multiple Interventions, with CC Score 3-4 |
| FD10D | Non-Malignant Gastrointestinal Tract Disorders with Multiple Interventions, with CC Score 0-2 |
| FD10E | Non-Malignant Gastrointestinal Tract Disorders with Single Intervention, with CC Score 9+ |
| FD10F | Non-Malignant Gastrointestinal Tract Disorders with Single Intervention, with CC Score 5-8 |
| FD10G | Non-Malignant Gastrointestinal Tract Disorders with Single Intervention, with CC Score 3-4 |
| FD10H | Non-Malignant Gastrointestinal Tract Disorders with Single Intervention, with CC Score 0-2 |
| FD10J | Non-Malignant Gastrointestinal Tract Disorders without Interventions, with CC Score 11+ |
| FD10K | Non-Malignant Gastrointestinal Tract Disorders without Interventions, with CC Score 6-10 |
| FD10L | Non-Malignant Gastrointestinal Tract Disorders without Interventions, with CC Score 3-5 |
| FD10M | Non-Malignant Gastrointestinal Tract Disorders without Interventions, with CC Score 0-2 |
| FD11A | Malignant Gastrointestinal Tract Disorders with Multiple Interventions, with CC Score 7+ |
| FD11B | Malignant Gastrointestinal Tract Disorders with Multiple Interventions, with CC Score 3-6 |
| FD11C | Malignant Gastrointestinal Tract Disorders with Multiple Interventions, with CC Score 0-2 |
| FD11D | Malignant Gastrointestinal Tract Disorders with Single Intervention, with CC Score 6+ |
| FD11E | Malignant Gastrointestinal Tract Disorders with Single Intervention, with CC Score 3-5 |
| FD11F | Malignant Gastrointestinal Tract Disorders with Single Intervention, with CC Score 0-2 |
| FD11G | Malignant Gastrointestinal Tract Disorders without Interventions, with CC Score 9+ |
| FD11H | Malignant Gastrointestinal Tract Disorders without Interventions, with CC Score 5-8 |
| FD11J | Malignant Gastrointestinal Tract Disorders without Interventions, with CC Score 3-4 |
| FD11K | Malignant Gastrointestinal Tract Disorders without Interventions, with CC Score 0-2 |
| FE01Z | Complex Therapeutic Endoscopic, Upper or Lower Gastrointestinal Tract Procedures |
| FE02A | Major Therapeutic Endoscopic, Upper or Lower Gastrointestinal Tract Procedures, 19 years and over, with CC Score 3+ |
| FE02B | Major Therapeutic Endoscopic, Upper or Lower Gastrointestinal Tract Procedures, 19 years and over, with CC Score 1-2 |
| FE02C | Major Therapeutic Endoscopic, Upper or Lower Gastrointestinal Tract Procedures, 19 years and over, with CC Score 0 |
| FE03A | Intermediate Therapeutic Endoscopic, Upper or Lower Gastrointestinal Tract Procedures, 19 years and over |
| FE10A | Endoscopic Insertion of Luminal Stent into Gastrointestinal Tract with CC Score 7+ |
| FE10B | Endoscopic Insertion of Luminal Stent into Gastrointestinal Tract with CC Score 4-6 |
| FE10C | Endoscopic Insertion of Luminal Stent into Gastrointestinal Tract with CC Score 1-3 |
| FE10D | Endoscopic Insertion of Luminal Stent into Gastrointestinal Tract with CC Score 0 |
| FE11A | Endoscopic, Sclerotherapy or Rubber Band Ligation, of Lesion of Upper Gastrointestinal Tract, with CC Score 9+ |
| FE11B | Endoscopic, Sclerotherapy or Rubber Band Ligation, of Lesion of Upper Gastrointestinal Tract, with CC Score 6-8 |
| FE11C | Endoscopic, Sclerotherapy or Rubber Band Ligation, of Lesion of Upper Gastrointestinal Tract, with CC Score 3-5 |
| FE11D | Endoscopic, Sclerotherapy or Rubber Band Ligation, of Lesion of Upper Gastrointestinal Tract, with CC Score 0-2 |
| FE12A | Endoscopic Insertion of Gastrostomy Tube, 19 years and over |
| FE13Z | Endoscopic Insertion of, Gastrojejunostomy or Jejunostomy Tube |
| FE20Z | Therapeutic Endoscopic Upper Gastrointestinal Tract Procedures, 19 years and over |
| FE21Z | Diagnostic Endoscopic Upper Gastrointestinal Tract Procedures with Biopsy, 19 years and over |
| FE22Z | Diagnostic Endoscopic Upper Gastrointestinal Tract Procedures, 19 years and over |
| FE30Z | Therapeutic Colonoscopy, 19 years and over |
| FE31Z | Diagnostic Colonoscopy with Biopsy, 19 years and over |
| FE32Z | Diagnostic Colonoscopy, 19 years and over |
| FE33Z | Therapeutic Flexible Sigmoidoscopy, 19 years and over |
| FE34Z | Diagnostic Flexible Sigmoidoscopy with Biopsy, 19 years and over |
| FE35Z | Diagnostic Flexible Sigmoidoscopy, 19 years and over |
| FE36Z | Diagnostic or Therapeutic, Rigid Sigmoidoscopy, 19 years and over |
| FE40Z | Therapeutic, Upper Gastrointestinal Tract Endoscopic Procedure with Colonoscopy, 19 years and over |
| FE41Z | Diagnostic, Upper Gastrointestinal Tract Endoscopic Procedure with Colonoscopy, with Biopsy, 19 years and over |
| FE42Z | Diagnostic, Upper Gastrointestinal Tract Endoscopic Procedure with Colonoscopy, 19 years and over |
| FE43Z | Therapeutic, Upper Gastrointestinal Tract Endoscopic Procedure with Sigmoidoscopy, 19 years and over |
| FE44Z | Diagnostic, Upper Gastrointestinal Tract Endoscopic Procedure with Sigmoidoscopy, with Biopsy, 19 years and over |
| FE45Z | Diagnostic, Upper Gastrointestinal Tract Endoscopic Procedure with Sigmoidoscopy, 19 years and over |
| FE50A | Wireless Capsule Endoscopy, 19 years and over |
| FF02A | Complex, Oesophageal, Stomach or Duodenum Procedures, 19 years and over, with CC Score 4+ |
| FF02B | Complex, Oesophageal, Stomach or Duodenum Procedures, 19 years and over, with CC Score 2-3 |
| FF02C | Complex, Oesophageal, Stomach or Duodenum Procedures, 19 years and over, with CC Score 0-1 |
| FF04A | Major, Oesophageal, Stomach or Duodenum Procedures, 19 years and over, with CC Score 7+ |
| FF04B | Major, Oesophageal, Stomach or Duodenum Procedures, 19 years and over, with CC Score 4-6 |
| FF04C | Major, Oesophageal, Stomach or Duodenum Procedures, 19 years and over, with CC Score 2-3 |
| FF04D | Major, Oesophageal, Stomach or Duodenum Procedures, 19 years and over, with CC Score 0-1 |
| FF05Z | Intermediate Upper Gastrointestinal Tract Procedures, 19 years and over |
| FF30A | Very Complex Large Intestine Procedures with CC Score 9+ |
| FF30B | Very Complex Large Intestine Procedures with CC Score 6-8 |
| FF30C | Very Complex Large Intestine Procedures with CC Score 3-5 |
| FF30D | Very Complex Large Intestine Procedures with CC Score 0-2 |
| FF31A | Complex Large Intestine Procedures, 19 years and over, with CC Score 9+ |
| FF31B | Complex Large Intestine Procedures, 19 years and over, with CC Score 6-8 |
| FF31C | Complex Large Intestine Procedures, 19 years and over, with CC Score 3-5 |
| FF31D | Complex Large Intestine Procedures, 19 years and over, with CC Score 0-2 |
| FF32A | Proximal Colon Procedures, 19 years and over, with CC Score 6+ |
| FF32B | Proximal Colon Procedures, 19 years and over, with CC Score 3-5 |
| FF32C | Proximal Colon Procedures, 19 years and over, with CC Score 0-2 |
| FF33A | Distal Colon Procedures, 19 years and over, with CC Score 3+ |
| FF33B | Distal Colon Procedures, 19 years and over, with CC Score 0-2 |
| FF34A | Major Large Intestine Procedures, 19 years and over, with CC Score 3+ |
| FF34B | Major Large Intestine Procedures, 19 years and over, with CC Score 1-2 |
| FF34C | Major Large Intestine Procedures, 19 years and over, with CC Score 0 |
| FF36Z | Intermediate Large Intestine Procedures, 19 years and over |
| FF40A | Major Anal Procedures, 19 years and over, with CC Score 1+ |
| FF40B | Major Anal Procedures, 19 years and over, with CC Score 0 |
| FF41A | Intermediate Anal Procedures, 19 years and over, with CC Score 3+ |
| FF41B | Intermediate Anal Procedures, 19 years and over, with CC Score 1-2 |
| FF41C | Intermediate Anal Procedures, 19 years and over, with CC Score 0 |
| FF42Z | Minor Anal Procedures |
| FF43Z | Minimal Anal Procedures |

*Following clinical review (CS 05/05/22) the following currencies did not represent activity on the wards of the district general hospital for the prospective study and were therefore EXCLUDED from the frequency weighted average calculation.*

| Currency | Currency Description |
| --- | --- |
|  |  |
| FF01A | Very Complex, Oesophageal, Stomach or Duodenum Procedures, 19 years and over, with CC Score 6+ |
| FF01B | Very Complex, Oesophageal, Stomach or Duodenum Procedures, 19 years and over, with CC Score 3-5 |
| FF01C | Very Complex, Oesophageal, Stomach or Duodenum Procedures, 19 years and over, with CC Score 0-2 |
| FF03A | Very Complex or Complex, Oesophageal, Stomach or Duodenum Procedures, 18 years and under, with CC Score 2+ |
| FF03B | Very Complex or Complex, Oesophageal, Stomach or Duodenum Procedures, 18 years and under, with CC Score 0-1 |
| FF10Z | Complex Surgical Procedures for Obesity |
| FF11Z | Major Surgical Procedures for Obesity |
| FF12Z | Sleeve Gastrectomy for Obesity |
| FF13Z | Gastric Band Procedures for Obesity |
| FF14Z | Adjustment of Gastric Band for Obesity |
| FF20A | Complex Small Intestine Procedures, 19 years and over, with CC Score 7+ |
| FF20B | Complex Small Intestine Procedures, 19 years and over, with CC Score 3-6 |
| FF20C | Complex Small Intestine Procedures, 19 years and over, with CC Score 0-2 |
| FF20D | Complex Small Intestine Procedures, 18 years and under |
| FF21A | Very Major Small Intestine Procedures, 19 years and over, with CC Score 8+ |
| FF21B | Very Major Small Intestine Procedures, 19 years and over, with CC Score 5-7 |
| FF21C | Very Major Small Intestine Procedures, 19 years and over, with CC Score 2-4 |
| FF21D | Very Major Small Intestine Procedures, 19 years and over, with CC Score 0-1 |
| FF22A | Major Small Intestine Procedures, 19 years and over, with CC Score 7+ |
| FF22B | Major Small Intestine Procedures, 19 years and over, with CC Score 4-6 |
| FF22C | Major Small Intestine Procedures, 19 years and over, with CC Score 2-3 |
| FF22D | Major Small Intestine Procedures, 19 years and over, with CC Score 0-1 |
| FF23A | Very Major or Major, Small Intestine Procedures, between 2 and 18 years, with CC Score 2+ |
| FF23B | Very Major or Major, Small Intestine Procedures, between 2 and 18 years, with CC Score 0-1 |
| FF23C | Very Major or Major, Small Intestine Procedures, 1 year and under, with CC Score 3+ |
| FF23D | Very Major or Major, Small Intestine Procedures, 1 year and under, with CC Score 1-2 |
| FF23E | Very Major or Major, Small Intestine Procedures, 1 year and under, with CC Score 0 |
| FF37A | Appendicectomy Procedures, 19 years and over, with CC Score 5+ |
| FF37B | Appendicectomy Procedures, 19 years and over, with CC Score 3-4 |
| FF37C | Appendicectomy Procedures, 19 years and over, with CC Score 1-2 |
| FF37D | Appendicectomy Procedures, 19 years and over, with CC Score 0 |
| FF37E | Appendicectomy Procedures, 18 years and under, with CC Score 3+ |
| FF37F | Appendicectomy Procedures, 18 years and under, with CC Score 1-2 |
| FF37G | Appendicectomy Procedures, 18 years and under, with CC Score 0 |
| FF47Z | Insertion of Neurostimulator for Treatment of Faecal Incontinence |
| FF48Z | Insertion of Neurostimulator Electrodes for Treatment of Faecal Incontinence |
| FF50A | Complex General Abdominal Procedures with CC Score 6+ |
| FF50B | Complex General Abdominal Procedures with CC Score 3-5 |
| FF50C | Complex General Abdominal Procedures with CC Score 0-2 |
| FF51A | Major General Abdominal Procedures, 19 years and over, with CC Score 10+ |
| FF51B | Major General Abdominal Procedures, 19 years and over, with CC Score 6-9 |
| FF51C | Major General Abdominal Procedures, 19 years and over, with CC Score 3-5 |
| FF51D | Major General Abdominal Procedures, 19 years and over, with CC Score 1-2 |
| FF51E | Major General Abdominal Procedures, 19 years and over, with CC Score 0 |
| FF51F | Major General Abdominal Procedures, between 2 and 18 years, with CC Score 1+ |
| FF51G | Major General Abdominal Procedures, between 2 and 18 years, with CC Score 0 |
| FF51H | Major General Abdominal Procedures, 1 year and under, with CC Score 2+ |
| FF51J | Major General Abdominal Procedures, 1 year and under, with CC Score 0-1 |
| FF52A | Intermediate Therapeutic General Abdominal Procedures, 19 years and over, with CC Score 3+ |
| FF52B | Intermediate Therapeutic General Abdominal Procedures, 19 years and over, with CC Score 1-2 |
| FF52C | Intermediate Therapeutic General Abdominal Procedures, 19 years and over, with CC Score 0 |
| FF52D | Intermediate Therapeutic General Abdominal Procedures, 18 years and under |
| FF53A | Minor Therapeutic or Diagnostic, General Abdominal Procedures, 19 years and over |
| FF53B | Minor Therapeutic or Diagnostic, General Abdominal Procedures, 18 years and under |
| FF60A | Complex Hernia Procedures with CC Score 5+ |
| FF60B | Complex Hernia Procedures with CC Score 3-4 |
| FF60C | Complex Hernia Procedures with CC Score 1-2 |
| FF60D | Complex Hernia Procedures with CC Score 0 |
| FF61A | Abdominal Hernia Procedures, 19 years and over, with CC Score 4+ |
| FF61B | Abdominal Hernia Procedures, 19 years and over, with CC Score 1-3 |
| FF61C | Abdominal Hernia Procedures, 19 years and over, with CC Score 0 |
| FF61D | Abdominal Hernia Procedures, 18 years and under |
| FF62A | Inguinal, Umbilical or Femoral Hernia Procedures, 19 years and over, with CC Score 6+ |
| FF62B | Inguinal, Umbilical or Femoral Hernia Procedures, 19 years and over, with CC Score 3-5 |
| FF62C | Inguinal, Umbilical or Femoral Hernia Procedures, 19 years and over, with CC Score 1-2 |
| FF62D | Inguinal, Umbilical or Femoral Hernia Procedures, 19 years and over, with CC Score 0 |
| FF62E | Inguinal, Umbilical or Femoral Hernia Procedures, between 2 and 18 years |
| FF62F | Inguinal, Umbilical or Femoral Hernia Procedures, 1 year and under |
| FF63A | Herniotomy Procedures, 2 years and over |
| FF63B | Herniotomy Procedures, 1 year and under |
| FF70A | Multiple Very Complex Gastrointestinal Tract Procedures, 19 years and over, with CC Score 7+ |
| FF70B | Multiple Very Complex Gastrointestinal Tract Procedures, 19 years and over, with CC Score 0-6 |
| FF70C | Multiple Very Complex Gastrointestinal Tract Procedures, 18 years and under |
