# Supplementary material for: A model-based cost-utility analysis of an automated notification system for deteriorating patients on general wards
Source: PLoS One. 2024 May 2;19(5):e0301643. doi: 10.1371/journal.pone.0301643 (PMC11065309; doi:10.1371/journal.pone.0301643)
Supplement: S2 Table — (DOCX) [file pone.0301643.s007.docx]

## **S3 Table. NHS Reference Costs Ward 2 (pulmonology) inclusion and exclusion.**

*Following clinical review (CS 05/05/22) the following currencies were considered plausible and included in the frequency weighted average calculation.*

| **Currency** | **Currency Description** |
| --- | --- |
|  |  |
| DZ09J | Pulmonary Embolus with Interventions, with CC Score 9+ |
| DZ09K | Pulmonary Embolus with Interventions, with CC Score 0-8 |
| DZ09L | Pulmonary Embolus without Interventions, with CC Score 12+ |
| DZ09M | Pulmonary Embolus without Interventions, with CC Score 9-11 |
| DZ09N | Pulmonary Embolus without Interventions, with CC Score 6-8 |
| DZ09P | Pulmonary Embolus without Interventions, with CC Score 3-5 |
| DZ09Q | Pulmonary Embolus without Interventions, with CC Score 0-2 |
| DZ10H | Lung Abscess-Empyema with Interventions, with CC Score 9+ |
| DZ10J | Lung Abscess-Empyema with Interventions, with CC Score 5-8 |
| DZ10K | Lung Abscess-Empyema with Interventions, with CC Score 0-4 |
| DZ10L | Lung Abscess-Empyema without Interventions, with CC Score 9+ |
| DZ10M | Lung Abscess-Empyema without Interventions, with CC Score 5-8 |
| DZ10N | Lung Abscess-Empyema without Interventions, with CC Score 0-4 |
| DZ11K | Lobar, Atypical or Viral Pneumonia, with Multiple Interventions, with CC Score 14+ |
| DZ11L | Lobar, Atypical or Viral Pneumonia, with Multiple Interventions, with CC Score 9-13 |
| DZ11M | Lobar, Atypical or Viral Pneumonia, with Multiple Interventions, with CC Score 0-8 |
| DZ11N | Lobar, Atypical or Viral Pneumonia, with Single Intervention, with CC Score 13+ |
| DZ11P | Lobar, Atypical or Viral Pneumonia, with Single Intervention, with CC Score 8-12 |
| DZ11Q | Lobar, Atypical or Viral Pneumonia, with Single Intervention, with CC Score 0-7 |
| DZ11R | Lobar, Atypical or Viral Pneumonia, without Interventions, with CC Score 14+ |
| DZ11S | Lobar, Atypical or Viral Pneumonia, without Interventions, with CC Score 10-13 |
| DZ11T | Lobar, Atypical or Viral Pneumonia, without Interventions, with CC Score 7-9 |
| DZ11U | Lobar, Atypical or Viral Pneumonia, without Interventions, with CC Score 4-6 |
| DZ11V | Lobar, Atypical or Viral Pneumonia, without Interventions, with CC Score 0-3 |
| DZ12C | Bronchiectasis with CC Score 8+ |
| DZ12D | Bronchiectasis with CC Score 5-7 |
| DZ12E | Bronchiectasis with CC Score 2-4 |
| DZ12F | Bronchiectasis with CC Score 0-1 |
| DZ13A | Cystic Fibrosis with CC |
| DZ13B | Cystic Fibrosis without CC |
| DZ14F | Pulmonary, Pleural or Other Tuberculosis, with Interventions |
| DZ14G | Pulmonary, Pleural or Other Tuberculosis, without Interventions, with CC Score 7+ |
| DZ14H | Pulmonary, Pleural or Other Tuberculosis, without Interventions, with CC Score 3-6 |
| DZ14J | Pulmonary, Pleural or Other Tuberculosis, without Interventions, with CC Score 0-2 |
| DZ15M | Asthma with Interventions |
| DZ15N | Asthma without Interventions, with CC Score 9+ |
| DZ15P | Asthma without Interventions, with CC Score 6-8 |
| DZ15Q | Asthma without Interventions, with CC Score 3-5 |
| DZ15R | Asthma without Interventions, with CC Score 0-2 |
| DZ16H | Pleural Effusion with Multiple Interventions, with CC Score 11+ |
| DZ16J | Pleural Effusion with Multiple Interventions, with CC Score 6-10 |
| DZ16K | Pleural Effusion with Multiple Interventions, with CC Score 0-5 |
| DZ16L | Pleural Effusion with Single Intervention, with CC Score 11+ |
| DZ16M | Pleural Effusion with Single Intervention, with CC Score 6-10 |
| DZ16N | Pleural Effusion with Single Intervention, with CC Score 0-5 |
| DZ16P | Pleural Effusion without Interventions, with CC Score 11+ |
| DZ16Q | Pleural Effusion without Interventions, with CC Score 6-10 |
| DZ16R | Pleural Effusion without Interventions, with CC Score 0-5 |
| DZ17L | Respiratory Neoplasms with Multiple Interventions, with CC Score 10+ |
| DZ17M | Respiratory Neoplasms with Multiple Interventions, with CC Score 6-9 |
| DZ17N | Respiratory Neoplasms with Multiple Interventions, with CC Score 0-5 |
| DZ17P | Respiratory Neoplasms with Single Intervention, with CC Score 10+ |
| DZ17Q | Respiratory Neoplasms with Single Intervention, with CC Score 6-9 |
| DZ17R | Respiratory Neoplasms with Single Intervention, with CC Score 0-5 |
| DZ17S | Respiratory Neoplasms without Interventions, with CC Score 13+ |
| DZ17T | Respiratory Neoplasms without Interventions, with CC Score 8-12 |
| DZ17U | Respiratory Neoplasms without Interventions, with CC Score 4-7 |
| DZ17V | Respiratory Neoplasms without Interventions, with CC Score 0-3 |
| DZ18D | Sleep Disorders Affecting Breathing, with Interventions, with CC Score 4+ |
| DZ18E | Sleep Disorders Affecting Breathing, with Interventions, with CC Score 0-3 |
| DZ18F | Sleep Disorders Affecting Breathing, without Interventions, with CC Score 4+ |
| DZ18G | Sleep Disorders Affecting Breathing, without Interventions, with CC Score 0-3 |
| DZ19H | Other Respiratory Disorders with Multiple Interventions |
| DZ19J | Other Respiratory Disorders with Single Intervention, with CC Score 5+ |
| DZ19K | Other Respiratory Disorders with Single Intervention, with CC Score 0-4 |
| DZ19L | Other Respiratory Disorders without Interventions, with CC Score 11+ |
| DZ19M | Other Respiratory Disorders without Interventions, with CC Score 5-10 |
| DZ19N | Other Respiratory Disorders without Interventions, with CC Score 0-4 |
| DZ20D | Pulmonary Oedema with Interventions |
| DZ20E | Pulmonary Oedema without Interventions, with CC Score 6+ |
| DZ20F | Pulmonary Oedema without Interventions, with CC Score 0-5 |
| DZ22K | Unspecified Acute Lower Respiratory Infection with Interventions, with CC Score 9+ |
| DZ22L | Unspecified Acute Lower Respiratory Infection with Interventions, with CC Score 0-8 |
| DZ22M | Unspecified Acute Lower Respiratory Infection without Interventions, with CC Score 13+ |
| DZ22N | Unspecified Acute Lower Respiratory Infection without Interventions, with CC Score 9-12 |
| DZ22P | Unspecified Acute Lower Respiratory Infection without Interventions, with CC Score 5-8 |
| DZ22Q | Unspecified Acute Lower Respiratory Infection without Interventions, with CC Score 0-4 |
| DZ23H | Bronchopneumonia with Multiple Interventions |
| DZ23J | Bronchopneumonia with Single Intervention, with CC Score 11+ |
| DZ23K | Bronchopneumonia with Single Intervention, with CC Score 0-10 |
| DZ23L | Bronchopneumonia without Interventions, with CC Score 11+ |
| DZ23M | Bronchopneumonia without Interventions, with CC Score 6-10 |
| DZ23N | Bronchopneumonia without Interventions, with CC Score 0-5 |
| DZ24J | Inhalation, Lung Injury or Foreign Body, with Multiple Interventions, with CC Score 10+ |
| DZ24K | Inhalation, Lung Injury or Foreign Body, with Multiple Interventions, with CC Score 0-9 |
| DZ24L | Inhalation, Lung Injury or Foreign Body, with Single Intervention, with CC Score 10+ |
| DZ24M | Inhalation, Lung Injury or Foreign Body, with Single Intervention, with CC Score 0-9 |
| DZ24N | Inhalation, Lung Injury or Foreign Body, without Interventions, with CC Score 14+ |
| DZ24P | Inhalation, Lung Injury or Foreign Body, without Interventions, with CC Score 10-13 |
| DZ24Q | Inhalation, Lung Injury or Foreign Body, without Interventions, with CC Score 6-9 |
| DZ24R | Inhalation, Lung Injury or Foreign Body, without Interventions, with CC Score 0-5 |
| DZ25G | Fibrosis or Pneumoconiosis, with Interventions, with CC Score 7+ |
| DZ25H | Fibrosis or Pneumoconiosis, with Interventions, with CC Score 0-6 |
| DZ25J | Fibrosis or Pneumoconiosis, without Interventions, with CC Score 10+ |
| DZ25K | Fibrosis or Pneumoconiosis, without Interventions, with CC Score 7-9 |
| DZ25L | Fibrosis or Pneumoconiosis, without Interventions, with CC Score 4-6 |
| DZ25M | Fibrosis or Pneumoconiosis, without Interventions, with CC Score 0-3 |
| DZ26G | Pneumothorax or Intrathoracic Injuries, with Multiple Interventions, with CC Score 6+ |
| DZ26H | Pneumothorax or Intrathoracic Injuries, with Multiple Interventions, with CC Score 0-5 |
| DZ26J | Pneumothorax or Intrathoracic Injuries, with Single Intervention, with CC Score 6+ |
| DZ26K | Pneumothorax or Intrathoracic Injuries, with Single Intervention, with CC Score 3-5 |
| DZ26L | Pneumothorax or Intrathoracic Injuries, with Single Intervention, with CC Score 0-2 |
| DZ26M | Pneumothorax or Intrathoracic Injuries, without Interventions, with CC Score 6+ |
| DZ26N | Pneumothorax or Intrathoracic Injuries, without Interventions, with CC Score 3-5 |
| DZ26P | Pneumothorax or Intrathoracic Injuries, without Interventions, with CC Score 0-2 |
| DZ27M | Respiratory Failure with Multiple Interventions, with CC Score 11+ |
| DZ27N | Respiratory Failure with Multiple Interventions, with CC Score 0-10 |
| DZ27P | Respiratory Failure with Single Intervention, with CC Score 11+ |
| DZ27Q | Respiratory Failure with Single Intervention, with CC Score 6-10 |
| DZ27R | Respiratory Failure with Single Intervention, with CC Score 0-5 |
| DZ27S | Respiratory Failure without Interventions, with CC Score 11+ |
| DZ27T | Respiratory Failure without Interventions, with CC Score 6-10 |
| DZ27U | Respiratory Failure without Interventions, with CC Score 0-5 |
| DZ28A | Pleurisy with CC Score 3+ |
| DZ28B | Pleurisy with CC Score 0-2 |
| DZ29G | Granulomatous, Allergic Alveolitis or Autoimmune Lung Disease, with Interventions |
| DZ29H | Granulomatous, Allergic Alveolitis or Autoimmune Lung Disease, without Interventions, with CC Score 5+ |
| DZ29J | Granulomatous, Allergic Alveolitis or Autoimmune Lung Disease, without Interventions, with CC Score 2-4 |
| DZ29K | Granulomatous, Allergic Alveolitis or Autoimmune Lung Disease, without Interventions, with CC Score 0-1 |
| DZ30Z | Chest Physiotherapy |
| DZ31Z | Cardiopulmonary Exercise Testing |
| DZ33Z | Hyperbaric Oxygen Treatment |
| DZ36Z | Bronchial Challenge Studies |
| DZ37A | Non-Invasive Ventilation Support Assessment, 19 years and over |
| DZ38Z | Oxygen Assessment and Monitoring |
| DZ42Z | TB Nurse Support |
| DZ45Z | Lung Volume Studies |
| DZ46Z | Respiratory Muscle Strength Studies |
| DZ49Z | Respiratory Nurse or AHP, Education or Support |
| DZ50Z | Respiratory Sleep Study |
| DZ51Z | Complex Tuberculosis |
| DZ52Z | Full Pulmonary Function Testing |
| DZ55Z | Bronchodilator Studies |
| DZ56Z | Carbon Monoxide Transfer Factor Test |
| DZ57Z | Oximetry or Blood Gas Studies |
| DZ58Z | Alveolar Carbon Monoxide Measurement or Smoking Cessation Support |
| DZ59Z | Airflow Studies |
| DZ60Z | Hypoxic (Altitude) or Hyperoxic (Shunt) Assessment |
| DZ65A | Chronic Obstructive Pulmonary Disease or Bronchitis, with Multiple Interventions, with CC Score 9+ |
| DZ65B | Chronic Obstructive Pulmonary Disease or Bronchitis, with Multiple Interventions, with CC Score 0-8 |
| DZ65C | Chronic Obstructive Pulmonary Disease or Bronchitis, with Single Intervention, with CC Score 9+ |
| DZ65D | Chronic Obstructive Pulmonary Disease or Bronchitis, with Single Intervention, with CC Score 5-8 |
| DZ65E | Chronic Obstructive Pulmonary Disease or Bronchitis, with Single Intervention, with CC Score 0-4 |
| DZ65F | Chronic Obstructive Pulmonary Disease or Bronchitis, without Interventions, with CC Score 13+ |
| DZ65G | Chronic Obstructive Pulmonary Disease or Bronchitis, without Interventions, with CC Score 9-12 |
| DZ65H | Chronic Obstructive Pulmonary Disease or Bronchitis, without Interventions, with CC Score 5-8 |
| DZ65J | Chronic Obstructive Pulmonary Disease or Bronchitis, without Interventions, with CC Score 0-4 |
| DZ65K | Chronic Obstructive Pulmonary Disease or Bronchitis, with length of stay 1 day or less, Discharged Home |
| DZ66Z | Complex Therapeutic Bronchoscopy |
| DZ67Z | Major Therapeutic Bronchoscopy |
| DZ68Z | Therapeutic Bronchoscopy |
| DZ69A | Diagnostic Bronchoscopy, 19 years and over |
| DZ70Z | Endobronchial Ultrasound Examination of Mediastinum |
| DZ71Z | Minor Thoracic Procedures |

*Following clinical review (CS 05/05/22) the following currencies did not represent activity on the wards of the district general hospital for the prospective study and were therefore EXCLUDED from the frequency weighted average calculation.*

| Currency | Currency Description |
| --- | --- |
|  |  |
| DZ01Z | Lung Transplant |
| DZ02H | Complex Thoracic Procedures, 19 years and over, with CC Score 6+ |
| DZ02J | Complex Thoracic Procedures, 19 years and over, with CC Score 3-5 |
| DZ02K | Complex Thoracic Procedures, 19 years and over, with CC Score 0-2 |
| DZ02L | Complex Thoracic Procedures, between 2 and 18 years |
| DZ02M | Complex Thoracic Procedures, 1 year and under |
| DZ32Z | Field Exercise Testing |
| DZ62A | Very Complex Thoracic Procedures with CC Score 6+ |
| DZ62B | Very Complex Thoracic Procedures with CC Score 3-5 |
| DZ62C | Very Complex Thoracic Procedures with CC Score 0-2 |
| DZ63A | Major Thoracic Procedures, 19 years and over, with CC Score 6+ |
| DZ63B | Major Thoracic Procedures, 19 years and over, with CC Score 3-5 |
| DZ63C | Major Thoracic Procedures, 19 years and over, with CC Score 0-2 |
| DZ63D | Major Thoracic Procedures, between 2 and 18 years |
| DZ63E | Major Thoracic Procedures, 1 year and under |
| DZ64A | Intermediate Thoracic Procedures, 19 years and over, with CC Score 6+ |
| DZ64B | Intermediate Thoracic Procedures, 19 years and over, with CC Score 3-5 |
| DZ64C | Intermediate Thoracic Procedures, 19 years and over, with CC Score 0-2 |
| DZ64D | Intermediate Thoracic Procedures, between 2 and 18 years |
| DZ64E | Intermediate Thoracic Procedures, 1 year and under |
