# Supplementary material for: A model-based cost-utility analysis of an automated notification system for deteriorating patients on general wards
Source: PLoS One. 2024 May 2;19(5):e0301643. doi: 10.1371/journal.pone.0301643 (PMC11065309; doi:10.1371/journal.pone.0301643)
Supplement: S3 Table — (DOCX) [file pone.0301643.s008.docx]

## **S4 Table. NHS Reference Costs Critical Care.**

*Serious events were calculated as the frequency weighted average of all activity codes in the group*

| **Currency** | **Currency Description** | **Activity** | **Unit Cost** | **Total Cost** |
| --- | --- | --- | --- | --- |
| XC01Z | Adult Critical Care, 6 or more Organs Supported | 9,785 | £ 2,330 | £ 22,795,140 |
| XC02Z | Adult Critical Care, 5 Organs Supported | 37,279 | £ 2,275 | £ 84,826,557 |
| XC03Z | Adult Critical Care, 4 Organs Supported | 123,347 | £ 2,160 | £ 266,466,570 |
| XC04Z | Adult Critical Care, 3 Organs Supported | 270,880 | £ 1,947 | £ 527,371,799 |
| XC05Z | Adult Critical Care, 2 Organs Supported | 333,957 | £ 1,704 | £ 569,077,527 |
| XC06Z | Adult Critical Care, 1 Organ Supported | 516,316 | £ 1,240 | £ 640,287,496 |
| XC07Z | Adult Critical Care, 0 Organs Supported | 35,030 | £ 1,087 | £ 38,086,370 |
