# Supplementary material for: A model-based cost-utility analysis of an automated notification system for deteriorating patients on general wards
Source: PLoS One. 2024 May 2;19(5):e0301643. doi: 10.1371/journal.pone.0301643 (PMC11065309; doi:10.1371/journal.pone.0301643)
Supplement: S4 Table — (DOCX) [file pone.0301643.s009.docx]

## **S5 Table. NHS Reference Costs Serious Events.**

*Serious events were calculated as the frequency weighted average of all non-elective activity codes in the group*

| **Currency** | **Currency Description** | Total  Non-elective activity |
| --- | --- | --- |
|  |  |  |
| EB10B | Actual or Suspected Myocardial Infarction, with CC Score 10-12 | 19,460 |
| EB10C | Actual or Suspected Myocardial Infarction, with CC Score 7-9 | 22,328 |
| EB10D | Actual or Suspected Myocardial Infarction, with CC Score 4-6 | 26,046 |
| EB10E | Actual or Suspected Myocardial Infarction, with CC Score 0-3 | 28,143 |
|  |  |  |
| DZ09J | Pulmonary Embolus with Interventions, with CC Score 9+ | 1,063 |
| DZ09K | Pulmonary Embolus with Interventions, with CC Score 0-8 | 541 |
| DZ09L | Pulmonary Embolus without Interventions, with CC Score 12+ | 5,256 |
| DZ09M | Pulmonary Embolus without Interventions, with CC Score 9-11 | 6,225 |
| DZ09N | Pulmonary Embolus without Interventions, with CC Score 6-8 | 9,776 |
| DZ09P | Pulmonary Embolus without Interventions, with CC Score 3-5 | 13,956 |
| DZ09Q | Pulmonary Embolus without Interventions, with CC Score 0-2 | 11,172 |
|  |  |  |
| DZ20D | Pulmonary Oedema with Interventions | 107 |
| DZ20E | Pulmonary Oedema without Interventions, with CC Score 6+ | 1,150 |
| DZ20F | Pulmonary Oedema without Interventions, with CC Score 0-5 | 466 |
|  |  |  |
| DZ27M | Respiratory Failure with Multiple Interventions, with CC Score 11+ | 89 |
| DZ27N | Respiratory Failure with Multiple Interventions, with CC Score 0-10 | 38 |
| DZ27P | Respiratory Failure with Single Intervention, with CC Score 11+ | 606 |
| DZ27Q | Respiratory Failure with Single Intervention, with CC Score 6-10 | 745 |
| DZ27R | Respiratory Failure with Single Intervention, with CC Score 0-5 | 327 |
| DZ27S | Respiratory Failure without Interventions, with CC Score 11+ | 2,394 |
| DZ27T | Respiratory Failure without Interventions, with CC Score 6-10 | 3,720 |
| DZ27U | Respiratory Failure without Interventions, with CC Score 0-5 | 3,071 |
|  |  |  |
| AA35A | Stroke with CC Score 16+ | 27,930 |
| AA35B | Stroke with CC Score 13-15 | 24,255 |
| AA35C | Stroke with CC Score 10-12 | 28,483 |
| AA35D | Stroke with CC Score 7-9 | 30,321 |
| AA35E | Stroke with CC Score 4-6 | 26,961 |
| AA35F | Stroke with CC Score 0-3 | 16,222 |

Continued

| **Currency** | **Currency Description** | **Total non-elective**  **Activity** |
| --- | --- | --- |
|  |  |  |
| WJ06A | Sepsis with Multiple Interventions, with CC Score 9+ | 2,757 |
| WJ06B | Sepsis with Multiple Interventions, with CC Score 5-8 | 2,686 |
| WJ06C | Sepsis with Multiple Interventions, with CC Score 0-4 | 1,098 |
| WJ06D | Sepsis with Single Intervention, with CC Score 9+ | 5,635 |
| WJ06E | Sepsis with Single Intervention, with CC Score 5-8 | 7,583 |
| WJ06F | Sepsis with Single Intervention, with CC Score 0-4 | 3,723 |
| WJ06G | Sepsis without Interventions, with CC Score 9+ | 41,890 |
| WJ06H | Sepsis without Interventions, with CC Score 5-8 | 93,413 |
| WJ06J | Sepsis without Interventions, with CC Score 0-4 | 76,709 |
|  |  |  |
| LA09J | General Renal Disorders with Interventions, with CC Score 6+ | 1,708 |
| LA09K | General Renal Disorders with Interventions, with CC Score 3-5 | 2,074 |
| LA09L | General Renal Disorders with Interventions, with CC Score 0-2 | 3,879 |
| LA09M | General Renal Disorders without Interventions, with CC Score 9+ | 1,978 |
| LA09N | General Renal Disorders without Interventions, with CC Score 6-8 | 3,582 |
| LA09P | General Renal Disorders without Interventions, with CC Score 3-5 | 10,351 |
| LA09Q | General Renal Disorders without Interventions, with CC Score 0-2 | 44,343 |
|  |  |  |
| EB05A | Cardiac Arrest with CC Score 9+ | 2,049 |
| EB05B | Cardiac Arrest with CC Score 5-8 | 1,053 |
| EB05C | Cardiac Arrest with CC Score 0-4 | 721 |
