# Supplementary material for: A model-based cost-utility analysis of an automated notification system for deteriorating patients on general wards
Source: PLoS One. 2024 May 2;19(5):e0301643. doi: 10.1371/journal.pone.0301643 (PMC11065309; doi:10.1371/journal.pone.0301643)
Supplement: S5 Table — (DOCX) [file pone.0301643.s010.docx]

## **S6 Table. Trimpoints used to calculate excess bed days.**

Weighted average of proportion of non-elective activity in each currency code.

Note on trimpoints: For each Health Resource Group (HRG), the trimpoint is calculated as the upper quartile length of stay for that HRG plus 1.5 times the inter-quartile range of length of stay, rounded to the nearest whole day. After the spell of treatment exceeds this number of days, a provider will receive payment for each additional day the patient remains in hospital. This is referred to as an excess bed day payment or a long stay payment.

|  | Trimpoint ^b&c^ |
| --- | --- |
| Ward 1 (gastroenterology) | 12 |
| Ward 2 (pulmonology) | 22 |
| Acute Myocardial Infarction | 19 |
| Pulmonary Embolus | 18 |
| Acute Pulmonary Oedema | 19 |
| Respiratory Failure | 27 |
| Stroke | 61 |
| Severe Sepsis | 26 |
| Acute Renal Failure | 13 |
| ICU (bed day) | n/a |
| Cardiopulmonary Arrest | 19 |
