# Supplementary material for: A model-based cost-utility analysis of an automated notification system for deteriorating patients on general wards
Source: PLoS One. 2024 May 2;19(5):e0301643. doi: 10.1371/journal.pone.0301643 (PMC11065309; doi:10.1371/journal.pone.0301643)
Supplement: S6 Table — (DOCX) [file pone.0301643.s011.docx]

## **S7 Table. Baseline characteristics of subgroup model populations.**

| Analysis | Description | N | Age (mean) | Female (%) | Gastro Ward (%) | COPD/ CFA (%) | No. of beds | LOS*_ward (mean days) control | LOS*_ward (mean days) intervention | SPOT cost (£/pt^) | IGS cost (£/pt^) |
| --- | --- | --- | --- | --- | --- | --- | --- | --- | --- | --- | --- |
| Basecase | All cases in Vital II RDB with complete data on: intervention, age, sex, ward, and NEWS on admission | 3787 | 68 | 0.52 | 0.44 | 0.27 | 54 | 8.90 | 8.62 | 1.52 | 19.98 |
| Subgroup Age | Older: 75-years + | 1601 | 83 | 0.56 | 0.41 | 0.28 | 54 | 9.84 | 9.49 | 1.68 | 20.67 |
|  | Younger: 17-74-years | 2186 | 57 | 0.49 | 0.46 | 0.26 | 54 | 8.18 | 7.93 | 1.39 | 19.44 |
| Subgroup NEWS | NEWS on admission 0-5 (mean 2.0) | 3015 | 67 | 0.52 | 0.52 | 0.17 | 54 | 8.63 | 8.55 | 1.47 | 19.93 |
|  | NEWS on admission 6+  (mean 7.6) | 722 | 71 | 0.54 | 0.14 | 0.60 | 54 | 9.75 | 8.79 | 1.66 | 20.11 |
| Subgroup Primary ICD | ICD 10  Diseases of respiratory system | 1720 | 69 | 0.52 | 0.11 | 0.50 | 54 | 8.87 | 8.01 | 1.51 | 19.50 |
|  | ICD 11  Diseases of digestive system | 944 | 63 | 0.50 | 0.95 | 0.07 | 54 | 9.01 | 9.02 | 1.54 | 20.30 |
|  | ICD ≠10\|11  NOT Diseases of respiratory or digestive system ^ | 871 | 76 | 0.53 | 0.56 | 0.11 | 54 | 8.79 | 9.21 | 1.50 | 20.44 |

*LOS, length of stay; ^£/pt: mean cost per patient. ^ICD Code (%) 0 Unknown (0.11); 1 Certain infectious and parasitic diseases (0.57); 2 Neoplasms (7.46); 4 Endocrine, nutritional, and metabolic diseases (2.30); 6 Diseases of the nervous system (5.17); 9 Diseases of the circulatory system (14.47); 100 “Others” (69.92).
