# Supplementary material for: A model-based cost-utility analysis of an automated notification system for deteriorating patients on general wards
Source: PLoS One. 2024 May 2;19(5):e0301643. doi: 10.1371/journal.pone.0301643 (PMC11065309; doi:10.1371/journal.pone.0301643)
Supplement: S7 Table — (DOCX) [file pone.0301643.s012.docx]

## **S8 Table. Patient Characteristics.**

|  | Control period | | | Intervention period | | |
| --- | --- | --- | --- | --- | --- | --- |
| Patient Characteristics (n=3787) |  | | |  | | |
| Number of patients | 1765 |  |  | 2022 |  |  |
| Ward 1 (gastroenterology) (n; % in arm) | 740 | 42% |  | 927 | 46% |  |
| Ward 2 (pulmonology) (n; % in arm) | 1025 | 58% |  | 1095 | 54% |  |
| Age in years (mean, 95%CI) | 67.91 | 67.15 | 68.68 | 68.29 | 67.55 | 69.04 |
| Age in years (median, IQR) | 70 | 58 | 81 | 72 | 59 | 82 |
| Male gender | 857 | 49% |  | 957 | 47% |  |
| Ward LOS (mean, 95%CI) | 8.90 | 8.51 | 9.28 | 8.62 | 8.24 | 9.00 |
| Ward LOS (median, IQR) | 7 | 4 | 11 | 6 | 3 | 11 |
| ICU LOS (mean, 95% CI) | 0.09 | 0.04 | 0.14 | 0.10 | 0.02 | 0.17 |
| NEWS on hospital admission | 3.17 | 3.04 | 3.30 | 3.14 | 3.02 | 3.26 |
| NEWS value on hospital admission Ward 1 | 1.76 | 1.61 | 1.90 | 1.93 | 1.79 | 2.07 |
| NEWS value on hospital admission Ward 2 | 4.19 | 4.01 | 4.37 | 4.16 | 3.99 | 4.33 |
| COPD and CFA diagnosis (n, %) | 486 | 28% |  | 535 | 26% |  |

Note. For patient characteristics of data used in subgroup analysis, see S7.
