# Supplementary material for: A model-based cost-utility analysis of an automated notification system for deteriorating patients on general wards
Source: PLoS One. 2024 May 2;19(5):e0301643. doi: 10.1371/journal.pone.0301643 (PMC11065309; doi:10.1371/journal.pone.0301643)
Supplement: S8 Table — (DOCX) [file pone.0301643.s013.docx]

## **S9 Table. Unadjusted frequency of serious adverse events and associated model probabilities.**

|  | Unadjusted number of events | | | | Adjusted* probability (Pr)  of event during inpatient stay | |
| --- | --- | --- | --- | --- | --- | --- |
|  | Control | | Intervention | | Control | Intervention |
|  | n |  | n |  |  |  |
| Acute Myocardial Infarction | 4 |  | 0 |  | 0.000001 | 0.000000 |
| Pulmonary Embolism | 3 |  | 2 |  | 0.000002 | 0.000001 |
| Acute Pulmonary Oedema | 4 |  | 1 |  | 0.000002 | 0.000000 |
| Respiratory Failure | 19 |  | 10 |  | 0.007226 | 0.003502 |
| Stroke | 0 |  | 0 |  | 0.000000 | 0.000000 |
| Severe Sepsis | 17 |  | 1 |  | 0.007389 | 0.000364 |
| Acute Renal Failure | 3 |  | 1 |  | 0.001510 | 0.000422 |
| ICU (n, mean) | 21 | 0.02 | 21 | 0.01 | 0.010189 | 0.009117 |
| Cardiopulmonary Arrest | 13 |  | 2 |  | 0.005795 | 0.000716 |
| Death (n, mean) | 114 | 0.065 | 130 | 0.064 | 0.048994 | 0.048209 |
| Patients with serious adverse events (n, mean) | 145 | 0.082 | 146 | 0.072 | 0.093271 | 0.066618 |
| Total number of patients with NO serious events | 1620 |  | 1876 |  |  |  |
| Events per patient with event (mean) | 1.37 |  | 1.15 |  |  |  |

Note. *Adjusted for baseline differences in age, sex, ward, and NEWS score on admission, using negative binominal regression.
