# Supplementary material for: A model-based cost-utility analysis of an automated notification system for deteriorating patients on general wards
Source: PLoS One. 2024 May 2;19(5):e0301643. doi: 10.1371/journal.pone.0301643 (PMC11065309; doi:10.1371/journal.pone.0301643)
Supplement: S9 Table — (DOCX) [file pone.0301643.s014.docx]

## **S10 Table. Results of Sensitivity and Scenario Analyses.**

| Analysis | Parameter description | Unit cost of monitoring  (£/Patient) | | Incremental cost (£) | Cost-**effectiveness**  ICER  (£/event avoided) |
| --- | --- | --- | --- | --- | --- |
|  |  | Intervention | Control |  |  |
| Base-case | Estimated product life 5-years  54 beds; 8.62 day episode  0.12 cableless | 19.98 | 1.52 | -12.17 | DOMINANT |
| Univariate Sensitivity | Estimated product life 10 years | 16.60 | 0.76 | -14.81 | DOMINANT |
|  | Estimated product life 15 years | 15.47 | 0.51 | -15. 70 | DOMINANT |
|  | Cableless sensor use = 0.00 | 6.77 | 1.52 | -25.48 | DOMINANT |
|  | Cableless sensor use = 1.00 | 114.30 | 1.52 | 82.80 | £3,107 |
| Threshold | £ IGS per patient per episode  (additional £12.08)  Equivalent of 0.23 Cableless (absolute) | 32.06 | n/a | -0.003 |  |
| Scenario_1 | Current use in district general hospital:  Cableless sensor use = 0%  Estimated product life 10 years | 3.39 | 0.76 | -28.12 | DOMINANT |
|  |  |  |  |  |  |
| Analysis | Parameter description | | Incremental lifetime costs (£) | Incremental QALY | Cost-**utility**  ICER  (£/QALY) |
| Base-case | Case mix QALE  No event population case mix by ward and COPD status | | -55.35 | 0.0287 | DOMINANT |
| Scenario | Lowest QALE & Highest £ for No Event  No event population = COPD | | 4.81 | 0.0121 | £369 |
|  | Highest QALE (case mix £) for No Event  No event population = Age sex matched QALE for population norm | | -55.35 | 0.0399 | DOMINANT |
