# Supplementary material for: A model-based cost-utility analysis of an automated notification system for deteriorating patients on general wards
Source: PLoS One. 2024 May 2;19(5):e0301643. doi: 10.1371/journal.pone.0301643 (PMC11065309; doi:10.1371/journal.pone.0301643)
Supplement: S10 Table — (DOCX) [file pone.0301643.s015.docx]

## **S11 Table. Net health benefit and net monetary benefit of alternative strategies [subgroups].**

| Strategy | | Inc.  QALY | Inc.  Cost (£) | ICER  (£/QALY) | Point estimate quadrant^ | NHB | NMB | Pop. NHB | Rank* |
| --- | --- | --- | --- | --- | --- | --- | --- | --- | --- |
| A | All patients | 0.0287 | -55.35 | -£1,926 | SE: Dominant | 0.0315 | £630 | £2,386,305 |  |
| B | Age≥75 | 0.0040 | -19.18 | -£4,812 | SE: Dominant | 0.0049 | £99 | £158,360 | 2 |
| C | Age<75 | 0.0880 | 224.30 | £2,548 | NE | 0.0768 | £1,536 | £3,358,100 | 1 |
| D | NEWS 0-2 | 0.0329 | 8.41 | £256 | NE | 0.0325 | £650 | £1,223,387 |  |
| E | NEWS 3+ | 0.0259 | -85.44 | -£3,300 | SE: Dominant | 0.0302 | £603 | £1,148,555 |  |
| J | NEWS <6 | 0.0375 | -14.45 | -£386 | SE: Dominant | 0.0382 | £764 | £2,302,633 | 1 |
| F | NEWS 6+ | -0.0161 | -52.59 | £3,269 | SW | -0.0135 | -£269 | -£194,372 | ≠CE |
| G | ICD 10 | 0.0705 | -13.86 | -£197 | SE: Dominant | 0.0712 | £1,424 | £2,449,402 | 1 |
| H | ICD 11 | 0.0781 | 337.03 | £4,313 | NE | 0.0613 | £1,226 | £1,157,004 | 2 |
| I | ICD Other | 0.0173 | 158.42 | £9,143 | NE | 0.0094 | £188 | £163,870 | 3 |

*Note.* ICER Incremental cost-effectiveness ratio (extra cost per extra unit of health effect); MHB Net Health Benefit; NMB Net Monetary benefit. Pop. NMB, Population Net Monetary Benefit = NMB*n, where n=subgroup size. ^ SE: South-east (IGS more health gain at lower cost) = DOMINANT; NE: North-east (IGS more health gain but more expensive); SW: South-west (IGS lower cost but poorer health outcome). * Rank 1=relatively more cost-effective at the £20,000 / QALY threshold; ≠CE not cost-effective.
