# Supplementary material for: A model-based cost-utility analysis of an automated notification system for deteriorating patients on general wards
Source: PLoS One. 2024 May 2;19(5):e0301643. doi: 10.1371/journal.pone.0301643 (PMC11065309; doi:10.1371/journal.pone.0301643)
Supplement: S1 File — (DOCX) [file pone.0301643.s016.docx]

**S1. Worked example of QALE Calculation.**

The proportional QALY shortfall, between the QALE for the population norm, and the event/condition QALE extracted from the literature, was calculated based on the age and sex of the secondary population using published Quality-Adjusted Life Expectancy Norms for the English Population, with 3.5% discount rate applied. (McNamara et al. 2023).

The proportional shortfall was then applied to and subtracted from the model population norm QALE (9.7732 based on age 68-years, 48% male) to calculate the aged, sex, standardised discounted QALE for each health state at discharge.

For example:

Economic model population: Aged 68-years, 52% female, remaining QALYs without disease/event = 9.77 (McNamara et al.)

Severe sepsis study population (Soares et al. 2012): Aged 63-years, 52% female

- QALYs with disease/event = 3.90 (Soares et al. 2012)
- QALYs without disease/event = 11.40 (McNamara et al.)
- QALY shortfall = 65.90%

Severe sepsis population in economic model = 9.77*(100-65.90)=3.33 remaining QALYs at discharge after surviving severe sepsis.
