# Supplementary material for: A model-based cost-utility analysis of an automated notification system for deteriorating patients on general wards
Source: PLoS One. 2024 May 2;19(5):e0301643. doi: 10.1371/journal.pone.0301643 (PMC11065309; doi:10.1371/journal.pone.0301643)
Supplement: Attachment — Submitted filename: VITAL II HE Response to Reviewers 23Nov23.docx [file pone.0301643.s017.docx]

Dr Christian P Subbe, DM, MRCP	Menai Bridge, the 23^rd^ of November 2023

21 Menai Quays

Menai Bridge

LL59 5DB

To the Associate Editor

Dr Dominic Luke Thorrington

PLOS ONE

**Re: PONE-D-23-27466**

**A model-based cost-utility analysis of an automated notification system for deteriorating patients on general wards**

Many thanks for the detailed and helpful comments from the reviewers that we tried to address to the best of our abilities.

We would like to apend the financial disclosure statement as requested:

 "Yes - the study was funded by Philips Healthcare. Bangor University received funding from Betsi Cadwaladr University Health Board, Wales, UK to complete this research, via a Research Service subcontract of “Specific Research Plan for Economic Evaluation of Philips IntelliVue Guardian Solution (IGS)” incorporated into the Observational Field Test/Post Market Study Agreement between Philips Medizin Systeme Böblingen GmbH and Betsi Cadwaladr University Local Health Board."

“The funder had not role in the study design, data collection and analysis but assisted with the preparation of the manuscript.”

**Comments to the Author**

1. Is the manuscript technically sound, and do the data support the conclusions?

The manuscript must describe a technically sound piece of scientific research with data that supports the conclusions. Experiments must have been conducted rigorously, with appropriate controls, replication, and sample sizes. The conclusions must be drawn appropriately based on the data presented.

Reviewer #1: Yes

Reviewer #2: Yes

Response by the authors: Many thanks.

2. Has the statistical analysis been performed appropriately and rigorously?

Reviewer #1: No

Reviewer #2: Yes

Response by the authors: We are unsure about the difference in opinion between Reviewer #1 and Reviewer #2. Without further detail we are unable to defend our approach. We are grateful to Reviewer #2 for support.

3. Have the authors made all data underlying the findings in their manuscript fully available?

The [PLOS Data policy](https://eur01.safelinks.protection.outlook.com/?url=http%3A%2F%2Fwww.plosone.org%2Fstatic%2Fpolicies.action%23sharing&data=05%7C01%7Cc.subbe%40bangor.ac.uk%7C7ded20932a2749914c4208dbcac22723%7Cc6474c55a9234d2a9bd4ece37148dbb2%7C0%7C1%7C638326707473113043%7CUnknown%7CTWFpbGZsb3d8eyJWIjoiMC4wLjAwMDAiLCJQIjoiV2luMzIiLCJBTiI6Ik1haWwiLCJXVCI6Mn0%3D%7C3000%7C%7C%7C&sdata=p%2FlhVt4z5gFx%2BDlfXSQ0ohL0aF%2BwZ9U0CiSerO9MVFs%3D&reserved=0) requires authors to make all data underlying the findings described in their manuscript fully available without restriction, with rare exception (please refer to the Data Availability Statement in the manuscript PDF file). The data should be provided as part of the manuscript or its supporting information, or deposited to a public repository. For example, in addition to summary statistics, the data points behind means, medians and variance measures should be available. If there are restrictions on publicly sharing data—e.g. participant privacy or use of data from a third party—those must be specified.

Reviewer #1: No

Reviewer #2: Yes

Response by the authors: Again: We are unsure about the difference in opinion between Reviewer #1 and Reviewer #2. The manuscript reports on an economic evaluation that utilised secondary data sources, including access to the Vital II Research Database, which is available on application to Betsi Cadwaladr University Health Board, Wales, UK. All parameters required to replicate the analysis are presented in the manuscript (revised Table 1). We believe that the reader of the manuscript will be able to replicate our analysis with the data supplied in the manuscript and the extensive appendices. We are grateful to Reviewer #2 for support.

4. Is the manuscript presented in an intelligible fashion and written in standard English?

PLOS ONE does not copyedit accepted manuscripts, so the language in submitted articles must be clear, correct, and unambiguous. Any typographical or grammatical errors should be corrected at revision, so please note any specific errors here.

Reviewer #1: Yes

Reviewer #2: Yes

Response by the authors: Many thanks.

5. Review Comments to the Author

Reviewer #1: The method is good but I do not agree with this topic. In a hospital with deteriorating patients both deployment of an electronic automated advisory vital signs and routine monitoring are needed. So it is crystal clear and does not need a study. You can not replace them but to have both. I can not accept this topic at all.

Response by the Authors: We thank Reviewer #1 for their time to review the manuscript. We believe that there is no evidence to suggest that deployment of electronic automated advisory monitoring is in routine clinical practice, and therefore the emergence of this technology requires robust economic evaluation to determine the case for cost-effectiveness.

The economic model is based on practice at a district general hospital in the UK, in which the automated advisory vital sign monitoring was/is deployed in less than 12% of beds (54/463 beds) as part of a post-market surveillance agreement. This intervention is not considered routine practice in the UK National Health Service (NHS).

We are not able to gauge why the reviewers experience might be substantially different from that of the authors of the VITAL II study: Professor Bellomo practises in Australia and is a globally leading academic in Intensive Care and Nephrology with over 180.000 citations of this work. Dr Chris Subbe is an experienced specialist in Acute, Respiratory & Critical Care Medicine who has first-hand insight into medical care in Germany, the United Kingdom, France, the United States and other healthcare systems: In our experience automated alarms to responders outside of a given ward area are not commonly used even in critical care areas and an absolute rarity outside of intensive care. While nearly all spot-check monitors on the market can be networked and connected to electronic health records this is in our experience currently still the exception, and the way that networked monitors are linked to responders is open to debate. Often the patient’s parent team might be the main and only team that can view signals and in human factors terms this is a risk. Beyond this we have found very limited evidence from the literature for automated alerts to Rapid Response teams. We have found no evidence for further analysis of which patient group would particularly benefit from networked alerts. We believe that the same holds true for the use of wearable monitors within hospital.

Reviewer #2: This paper presents a policy-relevant cost-effectiveness investigation utilizing peer-reviewed inputs and generally standard approaches. The analysis benefits both from a well-performed sensitivity analysis that allows for joint fluctuation in all parameters and from an array of sub-group analyses. The paper is well organized and the presentation is generally clear. Overall, this is a solid analysis.

Response by the authors: We thank reviewer #2 for their time to review the manuscript and their considered opinion.

This paper does contain at least one key flaw, but one that should be fixable in a fairly straightforward way. For all its elegance in probabilistically sampling an array of input parameters for its sensitivity analysis, the fundamental costing of the intervention is misconstrued in a manner that underestimates it. The authors estimate a per-patient use cost by taking the full annual cost (purchase price divided by useful life), and apportioning to an average patient the average proportion of a year a patient utilizes the monitoring system. This is incorrect in that it results in treating the time that the system is unused as costless, as though the NHS could only cover a percentage of the purchase price equal only to the percent of the year it will be actively used. Even as a distal payer, the full cost of the system will be passed to the NHS, and the true cost per patient will be very simply the annual cost divided by annual patients. Estimating this way will raise the estimated intervention cost per patient, and every ICER reliant upon it. However, the change will not alter findings to an extent that will alter the fundamental finding that the system will be well with in cost-effectiveness thresholds.

Response by the authors: We appreciate the comments by the reviewer. For the present study and in ongoing clinical practice we used one monitor for 6-8 patients. This meant that the monitor was based on the study ward and not available for other patients. This is based on the clinical utility of having a monitoring device available in areas with acutely unwell patients and the necessity to limit movement of devices for the purpose of infection prevention. We hope that this explains our approach. We have added to this effect to the manuscript: “Each spot-check monitor was used for a group of 6-8 co-located patients “

We appreciate the comment by the reviewer and the need to clarify our methodology. As the technology is exclusively for the ward bed being utilized (i.e., restricted by ward capacity, and assuming wards run at full capacity all year), the number of patients per year is calculated as 365 days / mean length of stay.

The reviewer’s suggestion therefore results in the same estimate as our original estimate, as demonstrated here:

Original estimate:

| Mean cost of technology per patient = | [(purchase price / product-life) + variable costs for 1-year] |  |
| --- | --- | --- |
|  | [(365/mean length of stay) * total number of beds  with automated notification system enabled] |  |

| Mean cost of technology per patient = | [(77,448.16/5)+30,201.94] | = 1.52 |
| --- | --- | --- |
|  | [(365/8.62)*54] |  |

Reviewer #2 suggestion:

| Mean cost of technology per patient = | [(purchase price / product-life) + variable costs for 1-year] |  |
| --- | --- | --- |
|  | Annual patients |  |

| Mean cost of technology per patient = | [(77,448.16/5)+30,201.94] | =1.52 |
| --- | --- | --- |

Where annual patients is: [(365/mean length of stay)* total number of beds with automated notification system enabled].

Whilst there is no change to the mean cost per patient, we have clarified the calculation in the manuscript, to highlight the annual number of patients, based on the number of ward beds, mean length of patient stay, and ward operating at 100% annual capacity (line 181 to183).

There is also another aspect of this paper that may or may not be correct, and discussion (if not revision) is warranted. It is very noteworthy that the (adjusted) difference in point estimates of the mortality between intervention and control (as presented in Table 2 and Table S9, roughly -.001) in are actually quite small and do not match the adjusted mortality reductions discussed in the Clinical Background section and reported in reference 14 (roughly -.016, AOR ~.079). Why is this? This difference certainly has a large influence on the estimate of the resulting effectiveness inputs. Discussion and possibly revision are warranted. This difference may be underselling the cost-effectiveness of the system.

Response by authors: many thanks for highlighting this item, as an item for review and clarification. The mortality difference in the clinical paper (reference 14) is an absolute mortality difference based on 4402 patients in the prospective before-and-after study. This difference is indeed comparatively small. For the clinical background section in the present manuscript, we have used relative mortality reduction.

Table 1 of the revised manuscript (previously Table 2 as referenced above) presents the adjusted point estimate for inpatient mortality based on 3787/4402 cases (with complete data; adjusted for baseline differences in intervention group, age sex, ward, base score on admission). In comparison to the clinical data set (n=4402) the economic evaluation (n=3787) had proportionally less deaths in the control arm, which when also adjusted for baseline differences resulted in a smaller difference (-0.001 adjusted probability in economic evaluation compared to -0.02 unadjusted rate in the clinical before-and-after study). We agree this may lead to a more conservative estimate of cost-effectiveness; however, this is balanced with a more robust analysis of available data (adjusted for baseline differences). As such, we have revised the manuscript to include discussion of this on lines 407 to 411:

“Finally, it is noted that the economic evaluation used a reduced sample of the before-and-after study (with complete NEWS score on admission n=3787/4402) and whilst adjusted probabilities used in the model are a robust reflection of available data, difference in point estimates of mortality between intervention and control of complete cases are more conservative than those reported in the effectiveness study (14), which may underestimate the cost-effectiveness of the intervention”.

These being my main concerns with the manuscript, a handful of other areas would benefit from further attention:

- On line 49, for clarity it would help if the phrasing were "remains cost-effective at 100% 'usage' " lest some readers infer a meaning of "remains cost effective at 100% probability."

Response by reviewers: Many thanks for the suggestion. We have followed the advice of the reviewer.

- The "deterioration" which triggers a notification should be more clearly defined in the clinical background section. I found myself turning to the underlying investigation (ref 14) to understand this. It was otherwise somewhat unclear at first read if a notification could meaningfully precede "events" and forestall them or simply more vigorously rallied clinical staff when an event was already occurring.

Response by reviewers: Many thanks for the request. We have added to the clinical background section. We hope that this is helpful and would value the feedback of the reviewer: “Deterioration was defined as a NEWS of 6 or more. A score of 6 led to the activation of a practitioner with critical care skills. The notification aims to prevent further deterioration to a degree that results in the need for admission to Intensive Care, death or cardio-pulmonary arrest.”

- On line 141 refers readers to S1 to understand how QALYs are estimated in the model. The example in S1 is well taken, However, the reader also needs to be referred to Table 2 for the sources of QALYs, OR they need to be also reported in S1. At least a sentence or two on how the authors searched for and/or selected these as their sources is also warranted. These are very key inputs.

Response by authors: Many thanks – we have added reference to the ‘Table 2’ toon line 141 and consequently revised the table numbering and position (Table 2 in the original submission is now Table 1 in the revised manuscript). We have also added footnote 4 to Table 1 (previously Table 2) to direct readers to S1 for the example. Line 132 of the revised manuscript now contains further details of the literature review, as requested. We have added a phrase in the methodology:

“Published economic evaluations were identified using UK National Institute for Health and Clinical Excellence (NICE) guidance and supplementary electronic searches of PubMed. Studies set in the UK, adopting a life-time horizon, reporting costs and QALYs for interventions/comparators that best reflected current practice, were selected.”

- Regarding lifetime resource use as discussed briefly in lines 146-150, this deserves more explanation. I see estimates with sources in Table 2, and the reader should be pointed there. Moreover, if the utilized estimates are directly reported in those sources, that should be mentioned in this paragraph. If the authors did analysis with those sources to arrive at the estimates in Table 2, that analysis should be described here.

Response by authors: As suggested, we have cross referenced the model parameters Table 1 (in revised manuscript, previously Table 2). We have also clarified the statement on post-discharge resource use:

“Post-discharge resource use was not available at a patient level and is captured within life-time costs (Table 1), calculated using secondary data [external to the VITAL II clinical study].”

The methods for obtaining and adjusting life-time costs are described under “Long-term costs” (line 210).

- Regarding the equations in lines 158-160, it is appreciated that the authors use as their main analysis a conservative 5-yr useful product life, and leave longer estimates to sensitivity analyses. For completeness, if training costs are a regular part of initiating use of this technology, that should also be included, or their omission mentioned as a limitation. Similarly, if maintenance costs are a regular cost of utilizing this technology, they should also be incorporated or their omission should be mentioned as a limitation.

Response by authors: Many thanks for drawing our attention to this concern. We have acknowledged this as a limitation in the revised manuscript lines 397 to 400. “The analysis did not account for maintenance costs of the electronic automated advisory vital signs monitoring and notification system, or routine spot-check monitoring. It was assumed the intervention would displace existing requirements; however, it may be reasonable to estimate a 10% increase to cover training and maintenance, in which case the intervention would remain dominant.”

- Regarding the footnotes in Table 1 and the related S2-5: The weighted average should mention what weights; I'm inferring "frequency weighted average," correct? Relatedly, in S5, is "activity" synonymous with "frequency"? And is S5 and input into S4? In which case I would re-order them.

Response by authors: We are grateful for the comment. Reviewer #2’s inference is correct; this is the frequency weighted average. We have revised the footnote of Table 2 (previously Table 1) and S2-5.

S2-5 column headings represent the terminology used in the original source data (Currency, Currency Description, Total Non-elective Activity). Where “Activity” is synonymous with frequency.

S4 is not the input into S5. S4 are the reference costs for ICU and S5 are the reference costs for adverse events. “ICU costs and serious adverse event activity costs were added to ward costs to provide a cost from admission to discharge/death (Table 1).”

- Lines 185 - 187 is the only place in the main text where discounting is discussed, and only with reference to the inpatient stays. Is discounting performed for lifetime (post-stay) costs and/or QALYs?

Response by authors: Many thanks for drawing our attention to this omission that has now been include in the revised manuscript: “Life-time costs and QALYs were discounted at a rate of 3.5%.”

- the Long-term costs section should at some point refer the reader clearly to Table 2.

Response by authors: The table has been referenced in the revised manuscript (Table 1 of revised version).

- Within Table 2, the "Distribution" column should also report either the relevant confidence interval or variance parameter around the point estimate in the 'point-estimate' column.

Response by authors: The distribution column has been updated accordingly and the footnote updated to aid interpretation. Many thanks for the suggestion.

- In Table 4, I note that though Quality-adjusted life expectancy is higher in the Intervention group, raw life expectancy is reported at about .014 life years shorter. Is this accurate? Also, why is this the only row in Table 4 to not have 95% central ranges? They should be included in this row as with the others if available.

Response by authors: Many thanks and apologies for the oversight. This was a transposing error, where intervention and control were mislabelled; however, on reflection Table 4 reports point estimates and PSA generated 95% central ranges. Life-years was a secondary calculation using estimated life expectancy from a published source and has therefore been removed from this table and the associated narrative.

- The note in Table S9 states the adjusted probabilities are adjusted for "age, sex, gender...." I wanted to check that it's accurate that the adjustment included both sex and gender distinctly construed? Or was it just one of them?

Response by authors: Again: Many thanks for drawing our attention to this error, this is an error and gender has been removed.

These detailed comments notwithstanding, the authors should be commended on a solid investigation, and I would expect that with revision this paper will reach a publishable form.

Response by the authors: We are very grateful to the reviewers for the detailed comments and the issues raised. We hope that the revised version will improve the experience of the reader and are looking forward to the feedback by the editorial team.

Yours truly,

Dr. Christian P Subbe, DM, FRCP

Consultant in Acute and Critical Care Medicine

Senior Clinical Lecturer, School of Medical Sciences

Bangor University, Wales, United Kingdom
